# IL-22 contributes to TGF-β1-mediated epithelial-mesenchymal transition in asthmatic bronchial epithelial cells

**DOI:** 10.1186/1465-9921-14-118

**Published:** 2013-11-01

**Authors:** Jill R Johnson, Michiyoshi Nishioka, Jamila Chakir, Paul-André Risse, Ibrahim Almaghlouth, Ahmad N Bazarbashi, Sophie Plante, James G Martin, David Eidelman, Qutayba Hamid

**Affiliations:** 1 Meakins-Christie Laboratories, McGill University, 3626 St. Urbain Street, Montréal, QC H2X 2P2, Canada; 2 Centre de recherche de l'institut universitaire de cardiologie et de pneumologie de Québec, Québec, Canada

## Abstract

**Background:**

Allergic asthma is characterized by airway inflammation in response to antigen exposure, leading to airway remodeling and lung dysfunction. Epithelial-mesenchymal transition (EMT) may play a role in airway remodeling through the acquisition of a mesenchymal phenotype in airway epithelial cells. TGF-β1 is known to promote EMT; however, other cytokines expressed in severe asthma with extensive remodeling, such as IL-22, may also contribute to this process. In this study, we evaluated the contribution of IL-22 to EMT in primary bronchial epithelial cells from healthy and asthmatic subjects.

**Methods:**

Primary bronchial epithelial cells were isolated from healthy subjects, mild asthmatics and severe asthmatics (n=5 patients per group). The mRNA and protein expression of epithelial and mesenchymal cell markers and EMT-associated transcription factors was evaluated following stimulation with TGF-β1, IL-22 and TGF-β1+IL-22.

**Results:**

Primary bronchial epithelial cells stimulated with TGF-β1 underwent EMT, demonstrated by decreased expression of epithelial markers (E-cadherin and MUC5AC) and increased expression of mesenchymal markers (N-cadherin and vimentin) and EMT-associated transcription factors. IL-22 alone had no effect on epithelial or mesenchymal gene expression. However, IL-22+TGF-β1 promoted the expression of some EMT transcription factors (Snail1 and Zeb1) and led to a more profound cadherin shift, but only in cells obtained from severe asthmatics.

**Conclusion:**

The impact of IL-22 on airway epithelial cells depends on the cytokine milieu and the clinical phenotype of the patient. Further studies are required to determine the molecular mechanism of IL-22 and TGF-β1 cooperativity in driving EMT in primary human bronchial epithelial cells.

## Introduction

Inflammation in allergic asthma reflects complex activation of the adaptive and innate immune systems [1]. The classical Th2 paradigm, which suggests that asthma is driven by interleukins (IL)-4, -5 and -13, is mostly associated with mild to moderate allergic asthma [2]. However, it fails to explain more severe forms of asthma that are often associated with the expression of Th1 cytokines such as interferon-γ and the more recently described Th17-associated cytokines IL-17 and IL-22 [3-6]. Strategies to treat asthma with targeted therapies against Th2 cytokines have not been successful or have been effective only in highly selected subsets of patients [7-10]. One explanation for this limited success may be that other T cell subsets play a role, such as Th17 cells, as they have been implicated in other inflammatory processes [11-13]. It is important to investigate these novel subsets of T cells at various stages of disease pathobiology. IL-22 is a Th17 cytokine predominantly expressed by memory CD4+ T cells with both reparative and pro-inflammatory properties [14]. However, the role of this mediator in asthma is poorly understood. The distribution of the IL-22 receptor suggests that IL-22 signals predominantly in non-immune cells [15] and therefore holds particular interest for certain features of asthma, including airway remodeling. A major feature of asthmatic airway remodeling is an increase in airway smooth muscle (ASM) mass that occurs in parallel with the severity of asthma [16-19], although the mechanisms responsible for this increase in ASM mass are still under investigation.

Epithelial-mesenchymal transition (EMT) is a mechanism that may account for the accumulation of subepithelial mesenchymal cells, thereby contributing to increased contractile cell mass and airway hyperresponsiveness. During EMT, epithelial cells lose their typical cell-cell junctions and cell polarity and acquire a more mesenchymal phenotype [20]. EMT is mainly characterized by the loss of epithelial markers such as cytokeratins, tight junction proteins and E-cadherin, the acquisition of mesenchymal markers such as vimentin and N-cadherin, and increased expression of the Snail, Twist and Zeb transcription factors [20]. A recent study in a mouse model of chronic house dust mite-driven allergic airway inflammation demonstrated the capacity of airway epithelial cells to acquire mesenchymal characteristics under these conditions [21]. This process was associated with increased airway smooth muscle mass and elevated TGF-β1 signalling in the lung. However, as evidence of EMT in this model was only observed at more severe stages of the disease, we were interested in ascertaining the contribution of cytokines expressed in severe asthma on the induction of EMT. As previous reports have demonstrated that IL-17A promotes EMT in airway epithelial cells in a TGF-β1-dependent manner [22] and contributes to airway remodeling in a mouse model of allergic airway inflammation [23], the aim of this study was to elucidate the *in vitro* impact of IL-22 in conjunction with TGF-β1 on the induction of a mesenchymal phenotype in primary human bronchial epithelial cells derived from healthy control subjects and patients with either mild or severe allergic asthma.

## Materials and methods

### Bronchial biopsies and immunohistochemistry

Tissue samples were provided from the Tissue Bank of the Respiratory Health Network of the FRSQ, MUHC site (http://swrsr.crc.chus.qc.ca/). Patients provided informed consent (approved by the local ethics committee) for bronchoscopy and the use of their samples. Biopsies were taken from the bronchi of healthy controls (n=5), mild asthmatics (n=5) and severe asthmatics (n=5) by fiberoptic bronchoscopy. Patient characteristics are provided in Table 1. The biopsies were fixed immediately in 10% formalin overnight, processed and embedded in paraffin to form blocks. Blocks were cut into 5 μm thick sections with a microtome and H&E staining was performed every 25–30 slides for the assessment of tissue morphology.

**Table 1:** Subject characteristics for bronchial biopsies primary bronchial epithelial cells

	**Healthy controls**	**Mild asthmatics**	**Severe asthmatics**
***Biopsies for immunohistochemical staining***			
Age (years)	30.3 ± 15.2	34.2 ± 13.1	50.2 ± 15.0
Sex	1 M/4 F	3 M/2 F	4 M/1 F
FEV1 (%)	105.8 ± 6.42	95.6 ± 13.4	56.8 ± 23.2
Atopy	2	5	5
Medication (μg/day)			*890 ± 690/200*
(inhaled corticosteroid/ long-acting β_2_ agonist)			
***Primary bronchial epithelial cells***			
Age (years)	28.8 ± 11.0	21.8 ± 1.5	49.8 ± 16.0
Sex	4 M/1 F	2 M/3 F	2 M/3 F
PC_20_ (mg/ml)	135.0 ± 68.8	4.2 ± 3.9	ND
FEV1 (%)	96.0 ± 13.7	95.0 ± 5.1	54.0 ± 17.0
Atopy	0	5	4 yes/1 no
Medication (μg/day)			*1050 ± 255/100*
(inhaled corticosteroid/ long-acting β_2_ agonist)			

### Immunohistochemistry

Biopsy sections were deparaffinized and rehydrated using xylene and a graded ethanol series (100%, 90% and 70% ethanol), followed by washing in PBS (three times for five minutes each). Antigen retrieval was performed by immersing the tissue sections in a pressure cooker filled with citrate buffer (pH 6.0) and heated for 15 minutes. Tissues were then permeabilized using 2% Triton-X for 30 minutes, then incubated with 5% hydrogen peroxide for 30 minutes to reduce the activity of endogenous peroxidases. Tissue sections were then blocked with blocking buffer (Dako) for 30 minutes followed by primary antibody incubation (polyclonal goat anti-human IL-22, 1:300, Abcam, catalog #ab18498) overnight at 4°C. Next, the secondary antibody (biotinylated polyclonal rabbit anti-goat IgG, 1:100, Dako, catalog #E0466) was added to the tissue for 45 minutes followed by another 45 minutes of incubation with HRP (1:100, Vector Laboratories, catalog # SA-5004). Washing was carried out after each step. Under a light microscope, DAB was added to each slide and staining development was observed to avoid over exposure. The reaction was stopped using deionized water. Sections were finally counterstained with hematoxylin (3 sec) followed by lithium carbonate (20 sec) and dehydrated in 90%-100% ethanol for 1 min then of xylene for 4 min. Slides were coverslipped using CytoSeal-60 Mounting Medium (Fisher Scientific). Slides were left to dry and visualized by light microscopy under 400× magnification. IL-22 positive cells were enumerated by counting the number of IL-22 positive cells (in brown) per mm^2^ of tissue.

### Epithelial cell culture

Epithelial cells were isolated from bronchial biopsies of healthy subjects, mild steroid-naïve asthmatics and severe asthmatic subjects. Subjects were recruited from the Asthma Clinic at l’Institut Universitaire de Cardiologie et de Pneumologie de Québec (Québec, QC, Canada). The ethics committee board approved the study and all subjects provided written informed consent. The asthmatic subjects were diagnosed according to the American Thoracic Society criteria [24]. The characteristics of the subjects are summarized in Table 1. Severe asthmatics were defined according to the ATS refractory asthma definition [25] and were on continuous treatment with high doses of inhaled CS and long-acting β_2_-agonists. Their asthma was stable with no exacerbations in the preceding four months. All subjects were non-smokers. Epithelial cells were isolated and characterized by immunofluorescence and flow cytometry using an anti-cytokeratin antibody from Calbiochem (San Diego, CA) as previously described [26,27]. Epithelial cells from asthmatic (mild n=5 and severe n=5) and normal (n=5) subjects were cultured in 6-well (for Western blot analysis) and 12-well (for RNA analysis) plates. Briefly, cells were stimulated with IL-22, TGF-β1 (both 10 ng/ml) or both cytokines together for a period of 3 (RNA analysis) or 5 (protein analysis) days.

### Cytokine stimulation

Cells were seeded onto 12- and 6-well plates as described above and grown in bronchial epithelial growth medium (BEGM, Lonza) supplemented with a bullet kit containing bovine pituitary extract, insulin, hydrocortisone, gentamycin/amphotericin, retinoic acid, transferrin, epinephrine and hEGF (Lonza). Additionally, medium was supplemented with heat-inactivated fetal bovine serum (10% in the growth medium, 1% in starvation medium). At confluence, cells were starved for 24 h (BEGM + 1% FBS), then treated daily with IL-22 (10 ng/ml), TGF-β1 (10 ng/ml) or a combination of IL-22 and TGF-β1 (both 10 ng/ml) for a period of 3 (RNA analysis) or 5 (protein analysis) days. The concentrations of IL-22 and TGF-β1 used for epithelial cell stimulation and the time points used for assessments were determined in a pilot study.

### Protein quantification and immunoblotting

Primary bronchial epithelial cells were lysed in 100 μL of lysis buffer (50 mM Tris–HCl pH 7.5, 1 mM EGTA, 1 mM EDTA, 1% (v/v) Triton X-100, 1 mM sodium orthovanadate, 5 mM sodium pyrophosphate, 50 mM sodium fluoride, 0.27 M sucrose, 5 mM sodium pyrophosphate decahydrate and protease inhibitors). Protein concentrations were quantified using the BCA Protein Assay Kit (ThermoScientific) according to the manufacturer’s instructions. Fifty micrograms of protein were boiled and separated on a 10% Pro-Pure Next Gel with Pro-Pure Running Buffer (Amresco, Solon, OH). After transferring proteins to nitrocellulose, membranes were blocked for 1 hour at room temperature in Odyssey Blocking Buffer (Li-Cor Biosciences, Lincoln, NE). Blots were then incubated with a goat anti-human IL-22 receptor antibody (1 μg/ml, AF2770, R&D Systems, Minneapolis, MN), a mouse anti-human E-cadherin antibody (1:600, ab1416, Abcam, Cambridge, MA), a rabbit anti-human N-cadherin antibody (1:1000, ab76057, Abcam) or a mouse anti-human GAPDH antibody (1:1500, MAB374, Millipore, Billerica, MA) overnight at 4°C. Donkey anti-goat IgG (1:15,000, #35518, DyLight™680, Thermo Scientific), donkey anti-goat IgG IRDye (1:15,000, #926-32214, Li-Cor) secondary antibody, goat anti-mouse IgG (1:15,000, #35518, DyLight™680, Thermo Scientific) secondary antibody or goat anti-rabbit IgG (1:15,000, #35571, DyLight™800, Thermo Scientific) secondary antibody was applied for 1 hour in the dark at room temperature (1:15,000). The signal was detected and quantified using a LI-COR Odyssey imaging system (LI-COR Biosciences). All samples were normalized to GAPDH and expressed as a ratio relative to the control sample.

### Real time RT-PCR

Total RNA was isolated from cultured primary bronchial epithelial cells and purified using the RNeasy Mini Kit (Qiagen, Toronto, Canada), supplemented with the RNase-Free DNase Set (Qiagen). cDNA was obtained using the QuantiTect Reverse Transcription cDNA Synthesis Kit (Qiagen), and the absence of DNA contamination was verified by excluding the reverse transcriptase from subsequent PCR reactions. cDNA was subjected to PCR using the Power SYBR Green PCR Master Mix (Applied Biosystems, Foster City, CA) to amplify human transcripts of E-cadherin, MUC5AC, N-cadherin, vimentin, Snail1, Snail2, Twist1, Twist2, Zeb1, Zeb2 and GAPDH using primers from Invitrogen (Table 2). Each PCR reaction was carried out as follows: 15 min at 95°C, 15 sec at 94°C, 30 sec at 60°C, and 30 sec at 72°C. Each cycle was repeated 40 times following the manufacturer's recommendations using a 7500 Fast Real-Time PCR System (Applied Biosystems) thermal cycler. Based on the comparative Ct method, gene expression levels were calculated and GAPDH was used as the housekeeping gene. Untreated control samples for each cell line were set to 100% and the fold change in expression in following treatment is represented in the bar graphs as mean ± standard error of the mean. Each condition was assessed based on three replicates with n=4-5 patients per group.

**Table 2:** Primers used for qPCR analysis

***Gene name***	***Target gene***	***Forward primer***	***Reverse primer***	***Amplicon size (nt)***
*Epithelial genes*				
**human E-cadherin**	**hCDH1**	GCCGAGAGCTACACGTTCA	GACCGGTGCAATCTTCAAA	88
**human mucin**	**hMUC5AC**	TTCCATGCCCGGGTACCTG	CAGGCTCAGTGTCACGCTCTT	200
*Mesenchymal genes*				
**human N-cadherin**	**hCDH2**	CTCCATGTGCCGGATAGC	CGATTTCACCAGAAGCCTCTAC	92
**human vimentin**	**hVIM**	GTTTCCCCTAAACCGCTAGG	AGCGAGAGTGGCAGAGGA	68
*Transcription factor genes*				
**human Snail1**	**hSNAI1**	GCTGCAGGACTCTAATCCAGA	ATCTCCGGAGGTGGGATG	84
**human Snail2**	**hSNAI2**	TGGTTGCTTCAAGGACACAT	GTTGCAGTGAGGGCAAGAA	66
**human Twist1**	**hTWIST1**	AAGGCATCACTATGGACTTTCTCT	GCCAGTTTGATCCCAGTATTTT	96
**human Twist2**	**hTWIST2**	TCTGAAACCTGAACAACCTCAG	CTGCTGTCCCTTCTCTCGAC	70
**human Zeb1**	**hZEB1**	GCTAAGAACTGCTGGGAGGAT	ATCCTGCTTCATCTGCCTGA	79-82
**human Zeb2**	**hZEB2**	AAGCCAGGGACAGATCAGC	CCACACTCTGTGCATTTGAACT	74
*Housekeeping gene*				
**human glyceraldehyde 3-phosphate dehydrogenase**	**hGAPDH**	AGCCACATCGCTCAGACAC	GCCCAATACGACGACCAAATCC	46

### Statistical analysis

Statistical analysis was performed using GraphPad Prism version 6. For statistical analyses between two groups, t-tests were used. Comparisons between more than two groups were performed by ANOVA, followed by a Tukey post-hoc test. A p-value of < 0.05 was considered to be statistically significant. Data are expressed as mean ± standard error of the mean.

## Results

### Increased expression of IL-22 and the IL-22 receptor in severe asthmatics

**Figure 1:**
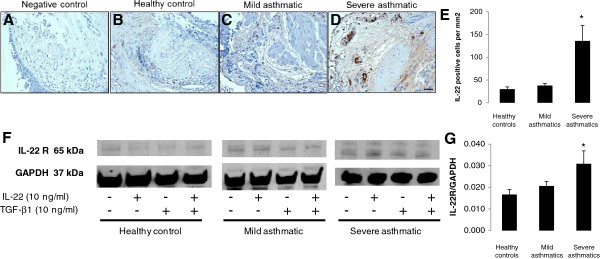
**IL-22 and IL-22 receptor expression levels increase in the airways of asthmatic subjects. (A-D)** Bronchial biopsies were obtained from healthy controls, mild asthmatics and severe asthmatics and stained (**A**, negative control) for IL-22 expression (in brown) by immunohistochemistry. Scale bar 50 μm. **(E)** The number of IL-22 positive cells was determined per mm^2^ of biopsy tissue. n=5 per group, *p<0.05 vs. healthy control. **(F)** Primary bronchial epithelial cells were obtained from healthy controls, mild asthmatics and severe asthmatics and assessed for IL-22 receptor expression by Western blot. Cells were allowed to grow to confluence, serum starved overnight and stimulated with IL-22, TGF-β1 (10 ng/mL each) or both cytokines for 5 days before Western blot analysis. **(G)** Expression levels of the IL-22 receptor in unstimulated cells were quantified relative to GAPDH expression. n=5 per group, *p<0.05 vs. healthy control.

Bronchial biopsies were obtained from healthy controls, mild asthmatics and severe asthmatics. Sections were stained by immunohistochemistry (negative control, Figure 1A) for the expression of IL-22 (Figure 1B-C), demonstrating a significantly greater influx of IL-22 expressing cells in the bronchi of severe asthmatics compared to mild asthmatics and healthy controls (Figure 1E; p < 0.05). The number of IL-22 positive cells was also normalized to the degree of inflammation in the biopsy using counts of IL-33 positive cells; the trends between groups and statistical significance remained consistent (data not shown).

Primary bronchial epithelial cells obtained from healthy controls, mild asthmatics and severe asthmatics were cultured and stimulated with IL-22, TGF-β1 or IL-22+TGF-β1 and assessed for their expression of the IL-22 receptor by immunoblotting (Figure 1F). Expression levels relative to the loading control (GAPDH) were assessed by densitometry, revealing significantly higher expression of the IL-22 receptor in unstimulated primary bronchial epithelial cells obtained from severe asthmatics compared to mild asthmatics and healthy controls (Figure 1G; p < 0.05). Stimulation with IL-22, TGF-β1 or IL-22+TGF-β1 *in vitro* for 5 days did not have a significant effect on the level of IL-22 receptor expression (data not shown).

### Exposure to TGF-β1 *in vitro* induces a mesenchymal phenotype in primary bronchial epithelial cells from mild and severe asthmatics

**Figure 2:**
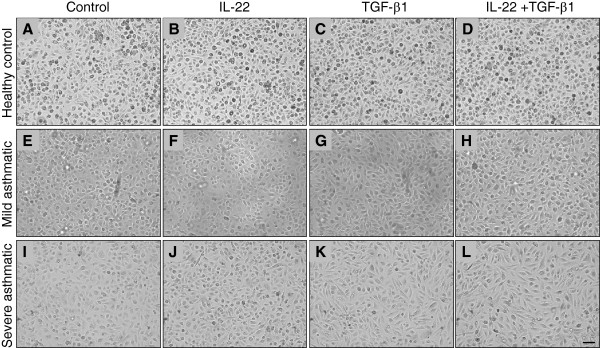
**TGF-β1 induces variable morphological changes in cultured primary epithelial cells.** Primary bronchial epithelial cells were obtained from healthy controls **(A-D)**, mild asthmatics **(E-H)** and severe asthmatics **(I-L)**, grown to confluence, serum starved overnight and stimulated with IL-22, TGF-β1 (10 ng/mL each) or both cytokines for 5 days before evaluating morphological changes. Scale bar 10 μm.

Cells were cultured for 5 days and treated with IL-22, TGF-β1 or IL-22+TGF-β1 (Figure 2). IL-22 alone did not have a discernible effect on the morphology of cultured primary bronchial epithelial cells taken from normal subjects or those obtained from patients with mild and severe asthma (Figure 2B, F, J). Conversely, an apparent morphological change was induced by TGF-β1, either with (Figure 2C, G, K) or without concomitant IL-22 treatment (Figure 2D, H, L). The most complete change to a mesenchymal phenotype was observed in cells obtained from severe asthmatics, with a high proportion of spindle-shaped cells seen in cultures from this group of patients following 5 days of TGF-β1 and IL-22+TGF-β1 stimulation (Figure 2K, L).

### TGF-β1 suppresses epithelial gene expression in primary bronchial epithelial cells

**Figure 3:**
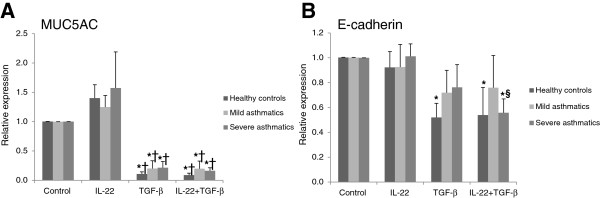
**Stimulation with TGF-β1 + IL-22 reduces MUC5AC and E-cadherin mRNA expression in primary bronchial epithelial cells.** Primary bronchial epithelial cells were obtained from healthy controls, mild asthmatics and severe asthmatics, grown to confluence, serum starved overnight and stimulated with IL-22, TGF-β1 (10 ng/mL each) or both cytokines for 5 days before evaluating E-cadherin **(A)** and MUC5AC **(B)** mRNA expression. Expression levels are relative to GAPDH expression. n=4-5 per group, *p<0.05 vs. control unstimulated cells, †p<0.05 vs. IL-22 stimulated cells, §p<0.05 vs. TGF-β1 stimulated cells.

In order to quantify the changes in epithelial gene expression in cultured primary bronchial epithelial cells, qPCR analysis was performed for the epithelial genes MUC5AC and E-cadherin, following 3 days of stimulation with IL-22, TGF-β1 or IL-22+TGF-β1 (Figure 3). MUC5AC expression was profoundly affected by stimulation with TGF-β1, with or without IL-22 stimulation; IL-22 stimulation in the context of TGF-β1 had no additional effect on the expression of MUC5AC (Figure 3A). E-cadherin mRNA expression was decreased in cells derived from normal subjects after 3 days of stimulation with TGF-β1 and IL-22+TGF-β1 (Figure 3B). No differences in E-cadherin mRNA expression were observed in cells derived from mild asthmatics following stimulation with TGF-β1 and IL-22+TGF-β1 (Figure 3B). Cells derived from severe asthmatics showed reduced relative expression of E-cadherin mRNA following stimulation with IL-22+TGF-β1 (Figure 3B).

### IL-22 stimulation does not affect mesenchymal gene expression in primary bronchial epithelial cells

**Figure 4:**
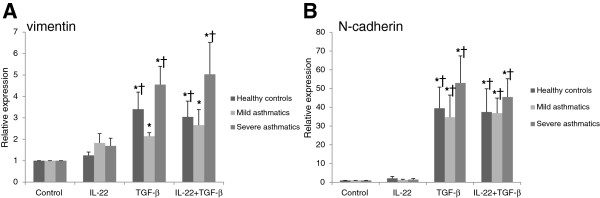
**TGF-β1, but not IL-22, increases the mRNA expression of mesenchymal genes in primary bronchial epithelial cells.** Primary bronchial epithelial cells were obtained from healthy controls, mild asthmatics and severe asthmatics, grown to confluence, serum starved overnight and stimulated with IL-22, TGF-β1 (10 ng/mL each) or both cytokines for 5 days before evaluating vimentin **(A)** and N-cadherin **(B)** mRNA expression. Expression levels are relative to GAPDH expression. n=4-5 per group, *p<0.05 vs. control unstimulated cells, †p<0.05 vs. IL-22 stimulated cells.

Changes in the relative expression of mesenchymal genes in cultured primary bronchial epithelial cells were evaluated by qPCR analysis for vimentin and N-cadherin mRNA, following 3 days of stimulation with IL-22, TGF-β1 or IL-22+TGF-β1 (Figure 4). As expected, TGF-β1 stimulation led to a significant increase in the expression of vimentin (Figure 4A) and N-cadherin (Figure 4B) in primary bronchial epithelial cells derived from healthy controls, mild asthmatics and severe asthmatics. IL-22 stimulation, either alone or in combination with TGF-β1, had no effect on the expression of vimentin or N-cadherin mRNA in primary bronchial epithelial cells from all three groups of subjects. The highest level of expression of both vimentin and N-cadherin was found in cells derived from severe asthmatics (Figure 4A, B).

### IL-22 cooperates with TGF-β1 in reducing E-cadherin protein expression in asthmatic primary bronchial epithelial cells

**Figure 5:**
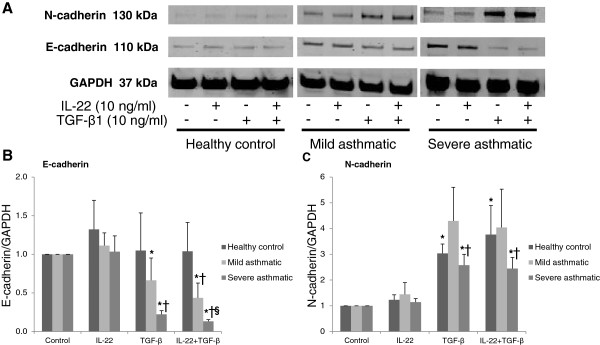
**IL-22 cooperates with TGF-β1 in reducing E-cadherin protein expression in asthmatic primary bronchial epithelial cells.** Primary bronchial epithelial cells were obtained from healthy controls, mild asthmatics and severe asthmatics, grown to confluence, serum starved overnight and stimulated with IL-22, TGF-β1 (10 ng/mL each) or both cytokines for 5 days before evaluating **(A)** E-cadherin and N-cadherin protein expression by Western blot. **(B, C)** Quantification of E-cadherin and N-cadherin expression levels is shown relative to GAPDH expression. n=4-5 per group, *p<0.05 vs. control unstimulated cells, †p<0.05 vs. IL-22 stimulated cells, §p=0.08 vs. TGF-β1 stimulated cells.

**Figure 6:**
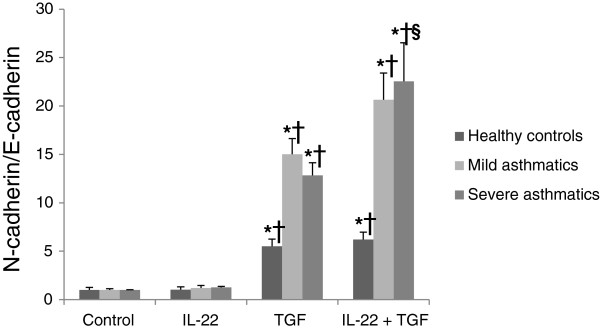
**Primary bronchial epithelial cells from severe asthmatics undergo a more profound cadherin switch in response to TGF-β1 and IL-22 stimulation.** Primary bronchial epithelial cells were obtained from healthy controls, mild asthmatics and severe asthmatics, grown to confluence, serum starved overnight and stimulated with IL-22, TGF-β1 (10 ng/mL each) or both cytokines for 5 days. **(A)** The switch in cadherin expression from E-cadherin (epithelial) to N-cadherin (mesenchymal) was assessed by Western blot. n=4-5 per group, *p<0.05 vs. control unstimulated cells, †p<0.05 vs. IL-22 stimulated cells, §p<0.05 vs. TGF-β1 stimulated cells.

Protein was collected from cultured cells after 5 days of treatment with IL-22, TGF-β1 or IL-22+TGF-β1 and evaluated by immunoblotting for the expression of E-cadherin and N-cadherin (Figure 5A). E-cadherin expression was decreased in response to TGF-β1 stimulation in cells derived from severe asthmatics, with a trend for a further decrease in E-cadherin expression with IL-22+TGF-β1 (Figure 5B; p=0.08). TGF-β1 stimulation induced a variable increase in N-cadherin expression in cells obtained from healthy controls, mild asthmatics and severe asthmatics relative to the housekeeping gene GAPDH (Figure 5C). The cadherin switch, indicative of epithelial-to-mesenchymal transition, was observed in all cells stimulated with TGF-β1 and IL-22+TGF-β1 for 5 days, although the most profound cadherin switch was observed in cells derived from severe asthmatics with an additive effect of IL-22+TGF-β1 in these cells (p < 0.05; Figure 6A).

### IL-22 cooperates with TGF-β1 in enhancing the expression of the EMT-associated transcription factors in primary bronchial epithelial cells from severe asthmatics

**Figure 7:**
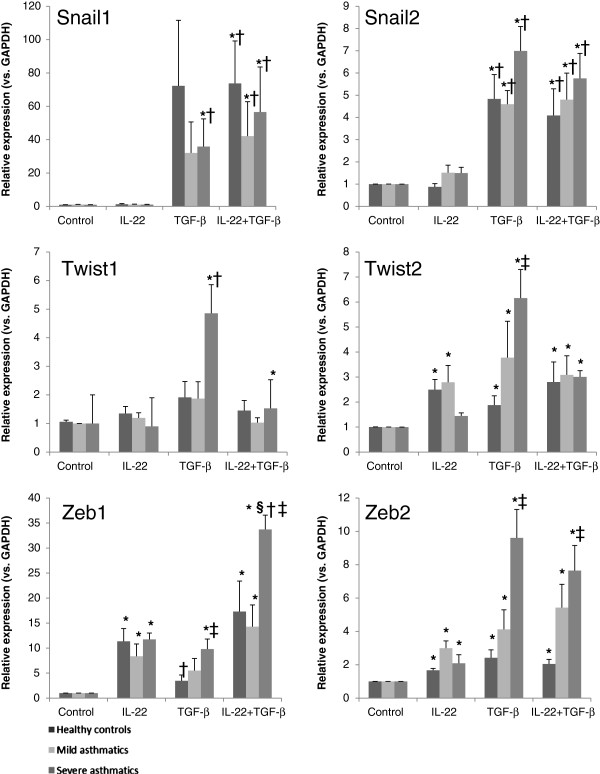
**IL-22 cooperates with TGF-β1 in promoting the expression of EMT-associated transcription factors.** Primary bronchial epithelial cells were obtained from healthy controls, mild asthmatics and severe asthmatics, grown to confluence, serum starved overnight and stimulated with IL-22, TGF-β1 (10 ng/mL each) or both cytokines for 5 days. The mRNA expression levels of transcription factor essential in epithelial-mesenchymal transition were assessed by qPCR. n=4-5 per group, *p<0.05 vs. control unstimulated cells, †p<0.05 vs. IL-22 stimulated cells, §p<0.05 vs. TGF-β1 stimulated cells, ‡p<0.05 vs. healthy control.

The transcriptional regulation of EMT in human primary bronchial epithelial cells was investigated following stimulation with IL-22, TGF-β1 or IL-22+TGF-β1 (Figure 7). The results of qPCR analysis of the mRNA expression levels of the EMT-associated transcription factors Snail1, Snail2, Twist1, Twist2, Zeb1 and Zeb2 show a significant upregulation of all transcription factors in response to stimulation with TGF-β1 (Figure 7A-F), most notably in cells derived from severe asthmatics. Interestingly, despite a significant increase in Twist1 and Twist2 expression following TGF-β1 stimulation, Twist transcription factor expression was relatively lower when cells were treated with IL-22+TGF-β1 compared to TGF-β1 alone (Figure 7C, D). Conversely, Snail1 and Zeb1 mRNA expression was significantly increased in cells from severe asthmatics treated with IL-22+TGF-β1 compared to TGF-β1 alone (Figure 7A, E). Stimulation with IL-22 alone led to a significant increase in the expression of the Zeb transcription factors in cells derived from all patient groups (Figure 7E, F).

## Discussion

The results of this study show that IL-22 and its receptor are highly expressed in the airways of severe asthmatics, and that bronchial epithelial cells from severe asthmatics are more sensitive to the effects of IL-22 stimulation in the context of TGF-β1 exposure, thus supporting a role for this cytokine in more severe, steroid refractory phenotypes of this disease.

It has become clear in recent years that different phenotypes of asthma are differentially regulated by cytokines. While Th2 cytokines are involved in milder forms of allergic asthma, Th17 cytokines (IL-17A, IL-17 F and IL-22) are more strongly associated with severe, difficult to treat asthma [3-6]. However, there is currently limited information on the role of Th17 associated cytokines, including IL-22, in human asthma. Zhao et al. demonstrated that the percentage of Th17 cells and plasma concentrations of IL-17 and IL-22 are increased in proportion to the severity of allergic airway disease [6]. In vitro, it has been shown that IL-22 promotes the proliferation and migration of airway smooth muscle cells [28,29]. It has also been shown that ovalbumin (OVA)-sensitized and challenged Balb/C mice express IL-22 in the lung, whereas this cytokine is undetectable in control animals [30]. Thus, it is likely that the co-expression of IL-22 along with other cytokines, for example IL-17A or TGF-β1, may have different effects than if IL-22 is expressed alone. In severe asthma, there is significantly higher expression of TGF-β1 compared to milder forms of asthma [31], suggesting the possibility that, in severe asthma, IL-22 may have different effects than in acute or mild disease because of the associated expression of TGF-β1. TGF-β1 is a potent promoter of EMT in airway epithelial cells [32]. Recently, it has been shown that TGF-β1-induced EMT in human bronchial epithelial cells is enhanced by IL-1β [33] and TNF-α [34], but the role of other cytokines such as IL-22 in the induction of EMT has not been explored.

The results from this study corroborate the findings of Zhao et al. [6] as IL-22 expression was predominantly detected in the subepithelial region of inflamed airways in severe asthma patients. As further support for the increased activity of IL-22 in severe asthma, primary bronchial epithelial cells obtained from severe asthmatics expressed significantly higher levels of the IL-22 receptor. Taken together, these results suggest that IL-22 expression and signaling is associated with severe allergic airway disease rather than milder forms of asthma. However, as some studies have demonstrated a tissue-protective role of IL-22 in terms of reducing the expression of proinflammatory cytokines such as IFN-γ [35] and enhancing barrier function [36], it was important to evaluate the impact of IL-22 stimulation on airway epithelial cells, both alone and in the context of stimulation with TGF-β1, a cytokine that is closely associated with severe asthma and tissue remodeling due to its role in the induction of EMT.

Previous studies have demonstrated that well-differentiated airway epithelial cell cultures from asthmatics undergo EMT more readily compared to control subjects, suggesting that epithelial repair in asthmatic airways is dysregulated [32], a finding which is supported by the results of the current study. Based on cellular morphology following 5 days of stimulation with TGF-β1, either with or without concomitant IL-22 stimulation, primary epithelial cells derived from patients with severe asthma underwent a more complete transition to a mesenchymal phenotype compared to cells from mild asthmatics and normal control subjects. This change from a typical epithelial cobblestone-like morphology to spindle-shaped mesenchymal cells driven by TGF-β1 is well-described in the literature, not only regarding airway epithelial cells in the context of asthma [17,32], but also in the context of tumor cell metastasis [37]. The results of this study show that the morphological change induced by TGF-β1 in airway epithelial cells is a factor of disease severity in the patients from whom the cells were derived, supporting previous studies [32], but covering a broader range of disease severity.

The switch from an epithelial to a mesenchymal phenotype was assessed by evaluating changes in the expression of epithelial E-cadherin and mesenchymal N-cadherin by qPCR, along with the expression of MUC5AC, an airway epithelial marker, and vimentin, a mesenchymal marker which is frequently investigated in studies on EMT [21]. TGF-β1 robustly decreased the expression of MUC5AC (by 80-90%) in primary bronchial epithelial cells from all subjects, demonstrating the loss of a characteristic airway epithelial cell marker under these conditions, although no further reduction in MUC5AC levels was observed when IL-22 was given to these cells along with TGF-β1. Conversely, TGF-β1 stimulation induced a milder (~50%) reduction in E-cadherin mRNA expression, which was only significant in cells from healthy control and severe asthmatics, suggesting that E-cadherin is more robustly expressed and tightly regulated than mucin genes under EMT conditions. IL-22 stimulation in the context of TGF-β1 exposure led to a further reduction in the expression of E-cadherin mRNA, although these changes were only statistically significant in cells derived from severe asthmatics. qPCR analysis was also performed for N-cadherin and vimentin to evaluate the impact of IL-22 and TGF-β1 stimulation on the expression of mesenchymal genes in bronchial epithelial cells. As expected, a significant upregulation in N-cadherin and vimentin mRNA was seen in the cells from all three patient groups following 3 days of stimulation with TGF-β1, while no effects of IL-22 were observed on the expression of mesenchymal genes, either when given alone or in combination with TGF-β1. These results demonstrate that, unlike TGF-β1, IL-22 is not a *bona fide* EMT-inducing cytokine, as it does not appear to induce a global change in epithelial and mesenchymal gene expression as observed in cells treated with TGF-β1. However, the further decrease in E-cadherin mRNA expression in severe asthmatic cells when IL-22 was added with TGF-β1 suggests that IL-22 may facilitate EMT in severe disease by further depressing E-cadherin expression.

This finding was supported by Western blot analysis of the cadherin switch in these cells, with significantly higher levels of N-cadherin and a virtual disappearance of E-cadherin seen in the cells from severe asthmatics following stimulation with TGF-β1. As seen on the mRNA level, a trend for a further decrease in E-cadherin expression was observed in severe asthmatic cells treated with both IL-22 and TGF-β1 compared to expression levels following TGF-β1 stimulation alone. This effect was more evident when the ratio of E-cadherin to N-cadherin was determined in these cells, as severe asthmatic cells demonstrated a more profound cadherin switch when IL-22 stimulation occurred in the context of TGF-β1 exposure. These results confirm that TGF-β1 potently suppresses the expression of epithelial adherens junction proteins in primary bronchial epithelial cells, and that concurrent stimulation with IL-22 contributes to this suppression, predominantly in cells taken from patients with severe asthma pathology. This finding is especially interesting given previous studies showing impaired intestinal epithelial barrier function in IL-22 deficient mice [36]. In the present study, treatment with IL-22 led to a slight but not significant increase in the expression of E-cadherin protein levels in healthy control cells; however, an assessment of barrier function in cultured airway epithelial cells was not within the scope of the present investigation.

The effects of TGF-β1 on epithelial and mesenchymal gene expression in human airway epithelial cells have been explored in a number of studies [21,32,33,38,39]. The results obtained in this study, with decreased expression of E-cadherin as well as increased expression of vimentin and N-cadherin, agree with these previous reports. However, the role of IL-22 in EMT, either alone or in the context of TGF-β1 stimulation, has not yet been investigated. This study provides novel results in that the combined impact of IL-22 with TGF-β1 was associated with an additive effect on the suppression of E-cadherin in primary bronchial epithelial cells, thus promoting the loss of adherens junctions in these cells, which has been previously described as an early event in the process of EMT [37]. It is important to highlight the fact that IL-22 mediated its most robust effects in the context of TGF-β1 stimulation in cells obtained from severe asthmatics. This result corroborates previous studies showing that asthmatic epithelial cells more readily progress through EMT [32], but provide novel insight into the mechanism by which this occurs. As IL-22 is highly expressed in severe asthmatics compared to mild asthmatics and normal control subjects, exposure to IL-22 *in vivo* may increase the sensitivity of these cells to EMT-promoting stimuli such as TGF-β1 *in vitro*. Further studies are certainly warranted to investigate the molecular mechanisms responsible for this, as well as the impact of other cytokines expressed in severe asthma, such as IL-17A, on the ability of bronchial epithelial cells to progress through EMT.

IL-22 mediates its signaling through a heterodimeric receptor composed of the IL-22R1 chain and the IL-10R2 chain [40]; downstream signaling is mediated predominantly via STAT3 [41]. Conversely, TGF-β1 signals through the type II TGF-β receptor (TGF-βRII), which then phosphorylates and activates signaling Smads such as Smad2, Smad3 and Smad4. Once activated, these Smads translocate to the nucleus to mediate their effects on the transcription of target genes [42]. To investigate the transcriptional regulation of EMT in primary bronchial epithelial cells stimulated with IL-22, TGF-β, and IL-22+TGF-β1, changes in the expression of EMT-associated transcription factors were investigated by qPCR. As expected, TGF-β1 stimulation alone potently upregulated the mRNA expression of all these transcription factors, most notably in cells derived from severe asthmatics. Costimulation with IL-22 and TGF-β1 had variable effects, with no change in the expression of Snail2 and Zeb2, a trend for a reduction in the expression of Twist1 and Twist2, and a significant increase in the expression of Snail1 and Zeb1 relative to expression levels following stimulation with TGF-β1 alone. Interestingly, the highest levels of Snail1 and Zeb1 were observed in cells obtained from severe asthmatics, with evidence of a synergistic effect of IL-22 and TGF-β1 on the mRNA expression of these key EMT-associated transcription factors in severe asthmatic bronchial epithelial cells, which may explain the profound cadherin switch observed in these cells. Previous studies have demonstrated that Snail1 forms a transcriptional repressor complex with Smad3 and Smad4 to promote EMT in epithelial cells; suppression of both Snail and Smad4 by siRNA potently suppressed the induction of EMT, supporting the key role played by these transcription factors in this process [43]. In the present study, concurrent stimulation of severe asthmatic bronchial epithelial cells with IL-22 and TGF-β1 led to a robust upregulation in Snail1 expression. This result may explain the effect of combined IL-22/TGF-β1 stimulation on E-cadherin repression in severe asthmatic cells, as this gene is highly sensitive to repression by the Snail1/Smad complex [43], whereas Twist transcription factors have been found to affect E-cadherin expression only indirectly [44].

Taken together, the results of this study suggest that the process of EMT as a factor contributing to the development of airway remodeling may only be clinically meaningful in patients with severe asthma. However, a strategy to inhibit the expression or signaling of cytokines that play a role in this process in milder stages of the disease may have a beneficial impact on lung structure and function by impeding this process. Further *in vivo* investigations are required to establish the effect of IL-22 inhibition on the progression of airway remodeling in chronic allergic asthma.

## Competing interests

The authors declare that they have no competing interests.

## Authors’ contributions

JRJ contributed to the design of the study, carried out cell culture experiments, performed PCR, analyzed the data, prepared the figures and drafted the manuscript; MN carried out cell culture experiments, performed PCR and immunoblotting and analyzed the data; JC provided primary cell cultures, contributed to the design of the study and contributed to manuscript preparation; PR contributed to the design of the study, carried out cell culture experiments, performed PCR and analyzed the data; IA carried out cell culture experiments and performed PCR; ANB carried out cell culture experiments and performed PCR; SP contributed to the design of the study; JM provided intellectual contributions and contributed to manuscript preparation; DE provided intellectual contributions and contributed to manuscript preparation; QH contributed to the design of the study, provided intellectual contributions, contributed to manuscript preparation and provided funding for the study. All authors read and approved the final manuscript.
